# Evaluation of an allied health professionals-led keratoconus service in a tertiary UK centre

**DOI:** 10.1038/s41433-026-04482-5

**Published:** 2026-05-06

**Authors:** Laura Elizabeth Boddy, ShinYi Woo, Pollyanna Murch, Dalia Said, Harminder Singh Dua

**Affiliations:** 1 Nottingham University Hospitals NHS Trust, Queen’s Medical Centre, Nottingham, UK; 2 Academic Ophthalmology, AU1, Mental Health and Clinical Neuroscience, School of Medicine, University of Nottingham, Nottingham, UK

**Keywords:** Health services, Therapeutics

## Abstract

**Background:**

Keratoconus is an ectatic corneal disorder, which requires regular monitoring for early detection of progression and prompt treatment with collagen cross-linking (CXL) to stabilise the condition. This conflicts with the demand on corneal specialists to review acute referrals into the corneal service, delaying diagnosis and treatment. In this study, we examined the outcomes of an Allied Health Professional (AHP)-led keratoconus service for routine workup, monitoring, performing CXL and follow-up thereafter.

**Methods:**

Refraction, topography and CXL operative details were taken from electronic patient records. Statistical analysis was performed in R^®^ (R Foundation for Statistical Computing, Vienna, Austria). 82 eyes had CXL performed by a nurse practitioner (NP) and 42 eyes by ophthalmologists. Pre- and post-operative evaluation were done by optometrists.

**Results:**

In total, 271 eyes of 177 patients were included. 92 patients (128 eyes) were consented and underwent CXL. 85 patients (143 eyes) where CXL was not indicated, were monitored routinely. All patients had baseline refraction and topography. Patients were reviewed at 12 and 24 months. All had topography at every visit. 71/72 eyes (98.61%) had refraction at 12 months and 57/58 eyes (91.28%) had refraction at 24 months. Patients who were operated on by NP found improvements in visual acuity at 12 months and 24 months (*p* < 0.05, Mann–Whitney U Test).

**Conclusion:**

The AHP-led keratoconus service delivered satisfactory outcomes and can be an efficient means of dealing with these patients, complementing specialist corneal services in the NHS.

## Introduction

Keratoconus is the most common bilateral but often asymmetric corneal ectatic disorder, which predominantly manifests in young adults often with rapid progression, increasing irregular astigmatism and deterioration of vision [1–5]. Disease onset is typically within adolescence with gradual progression [6]. Its aetiology is multifactorial with eye rubbing implicated as a common risk factor theorised to cause mechanical breakdown of bonds between collagen fibres and the extracellular matrix [7]. Other identified risk factors include a history of atopy (e.g. asthma, eczema, allergy), a positive family history and geographical location [8].

Management is dependent upon disease severity at presentation and documented progression [9, 10]. Early keratoconus is managed with routine monitoring and refractive correction with glasses. Rigid gas-permeable lenses are suitable for patients with astigmatism that cannot be corrected with glasses. Severe or complicated disease requires corneal transplantation, namely a deep anterior lamellar keratoplasty (DALK) or a penetrating keratoplasty (PKP) to restore vision [9, 10].

Collagen cross-linking (CXL) remains the only treatment that slows or halts disease progression [11]. CXL strengthens the anterior corneal stroma by exposing the corneal stroma to riboflavin (vitamin B2), which is activated by exposure to UVA light. This induces covalent bond formation between collagen fibres through 2 pathways: an oxidation pathway that forms riboflavin free radical species and a glycosylation pathway [11].

Keratoconus patients need corneal topography, visual acuity and refractive error monitored to detect progression, which would indicate prompt intervention by CXL. Patients are typically monitored until the disease stabilises for a minimum of 5 years. Post-CXL monitoring includes assessment of epithelial healing, presence of haze or infection, regular refraction and topography, all of which creates a high demand on the corneal subspecialist clinics generating backlogs and delays.

An emerging change in ophthalmic practice in the United Kingdom (UK) is the utilisation of allied health professionals (AHPs) such as optometrists and nurse practitioners (NPs) to perform specialised tasks [12]. These AHPs have extended their skill sets to include independent prescribing capabilities and higher certification in corneal disease. The higher qualifications enable optometrists and NPs to diversify their skills and review patients with guidance or advice from consultant ophthalmologists. In this retrospective study, we analysed the outcomes of keratoconus patients in an AHP-led keratoconus service, from diagnosis to CXL and follow-up, at a single tertiary ophthalmic unit in the UK.

## Methods

### Participants

This study was registered as a service evaluation and quality improvement project (Audit ID 23–586 C). Participants were identified from patients referred to a single AHP-led keratoconus clinic at the Queen’s Medical Centre, Nottingham. Keratoconus was confirmed based on slit-lamp examination for signs suggestive of keratoconus such as Vogt Striae, Fleischer ring, Munson sign, and corneal topography. Exclusion criteria included other associated corneal pathologies such as scars, and patients who have undergone CXL historically before referral to the keratoconus service.

### Overview of the service structure

The service operates with a single optometrist experienced in assessing corneal pathologies. Each clinic session has six available clinic appointments and operates alongside a consultant-led corneal clinic. During a clinic appointment, an optometrist would carry out the following activities: an ophthalmic history, slit-lamp examination, subjective refraction, and corneal topography. These investigations would be interpreted by the optometrist, who will generate an initial management plan. This plan would be discussed with the corneal consultant in the clinic and the management plan mutually agreed. Patients were then scheduled for further monitoring for any evidence of progression, listed for corneal cross-linking or listed for a corneal graft.

### Training and accreditation

The entire service was protocol driven. The protocol to cover the entire patient pathway was developed by two corneal consultants, adapted from a similar model at another UK tertiary centre and modified to local conditions. This was approved through the local governance procedures and signed off for use. The AHPs were given appropriate training and education on the equipment, tests and procedures. The optometrist initially discussed every case, pre- and post-operatively (for 6 months) with the consultant and the NP performed 10 cases under direct consultant supervision: before being accredited. However, they have direct access to a corneal consultant for every clinic post accreditation.

### Collagen cross linking

The corneal cross-linking performed at the Queen’s Medical Centre follows an accelerated CXL protocol within a dedicated treatment suite. A small proportion of cases were undertaken by ophthalmologists in either a treatment suite or in ophthalmic theatres.

A standard epi-off procedure was used. Briefly, the central corneal epithelium was debrided using 20% alcohol, riboflavin 0.1% was applied to the exposed stroma every 2 min for 10 min followed by exposure to 7.20 J/cm2 UVA light over 12 min [11]. A bandage contact lens was applied at the end of the procedure. Post-operative medication included prednisolone acetate 0.5% drops (Predsol Minims, Bausch & Lomb U.K Limited) and levofloxacin preservative-free drops (Oftaquix, Santen UK Limited). Initial follow-up was at 1-week post-CXL and subsequently at 1, 3, 6, 12, and 24 months.

Patients where CXL was not indicated were similarly followed up for evidence of progression.

### Data collection

Patient demographic data was obtained from hospital electronic patient records (MediSIGHT Live). Patient age, sex, history of ophthalmic surgery, past ophthalmic history and medication history were recorded for each patient.

The refractive status of the eyes was determined using subjective refraction. Refraction data was obtained from either electronic patient records (mediSIGHT Live) or digitally scanned clinic notes and referral letters (DHR Unity).

Corneal topography was measured using the Oculus Pentacam AXL®. Topographic values collected to assess for disease progression were keratometry of the flat meridian (K1), keratometry of the steepest meridian (K2), maximum keratometry (Kmax), Pachymetry, and Posterior Float. Higher-order aberrations (HOAs) were obtained for the anterior and total cornea using wavefront analysis. The Belin ABCD progression analysis was examined for each visit.

Data from patients undergoing CXL was collected at their respective treatment and follow-up appointments. For patients not undergoing CXL, data from their first and last visits was collected, for this study.

### Statistical analysis

R® statistical software (R Foundation for Statistical Computing, Vienna, Austria) was used to analyse the data [13]. Normality was assessed using a Shapiro-Wilk test of normality. Mann–Whitney U-Tests were performed to assess difference in clinical values in the CXL and monitoring groups at different time points. Chi-squared tests were used to assess for significance in the number of complications following CXL by AHP and ophthalmologists.

## Results

**Fig. 1:**
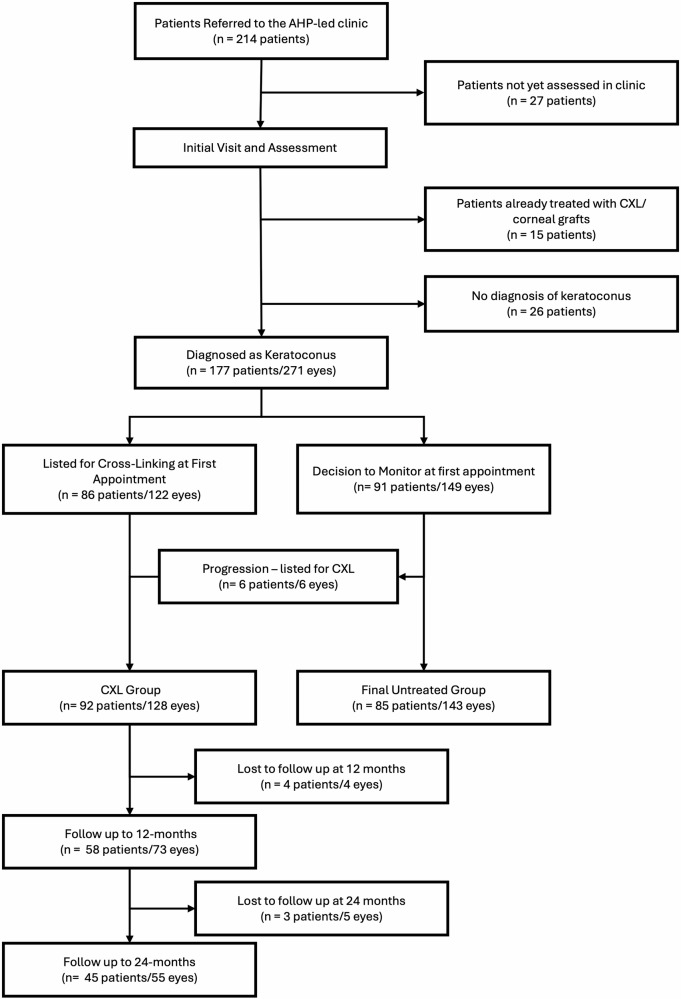
Flow diagram showing the number of patients from point of referral to final follow-up point.

Data was obtained for 177 patients (271 eyes). This included 128 eyes of 92 patients treated with CXL (CXL group) and 143 eyes of 85 patients that did not require CXL (untreated group). Flow diagram of the number of patients at each stage of management is shown in Fig. 1.

### Patient demographics

The median age at presentation for the CXL group was 24.50 years (24.05–26.01 95% Confidence Interval (CI)) and 30.00 years (28.68–31.61, 95% CI) for the untreated group. In the CXL group 26/86 (30.23%) patients were female, and 54/121 (44.63%) were right eyes. Fifty-one eyes of 36 patients wore Rigid Gas Permeable Lenses. In the untreated group, 28/85 (32.94%) patients were female and 71/147 (48.30%) eyes were right eyes. Forty eyes of 24 patients in the untreated group wore Rigid Gas Permeable Contact Lenses. Systemic and ocular past medical history of the 2 groups is listed in Table 1.

**Table 1:** List of associated medical conditions within the CXL and Monitoring groups.

*CXL Group*				*Monitoring Group*			
*Ophthalmic n* = *128*		*Systemic n* = *92*		*Ophthalmic n* = *143*		*Systemic n* = *85*	
Dry Eye	4	Hayfever	17	Dry Eye	5	Eczema	5
Glaucoma	3	Eczema	7	Corneal Scarring	4	Hayfever	4
Corneal Dystrophy	1	Asthma	5	Blepharitis	2	Idiopathic Intracranial Hypertension	1
Strabismus surgery	1	Psoriasis	2	Exposure Keratopathy	1		
		Diabetes	1	Acute Hydrops	1		
				Episcleritis	1		

### Analysis of CXL group at 12-months and 24-months

Seventy-three (57.03%) eyes were reviewed at 12 months and fifty-five (42.97%) eyes were reviewed at 24-months. All patients had baseline visual acuity, refraction and corneal topography performed prior to CXL. Seventy-one eyes (98.61%) had refraction performed at their 12-month follow-up. Fifty-seven eyes (98.28%) had refraction performed at their 24-month follow-up. All eyes had corneal topography measured at 12- and 24-months follow-up. Patients in this group have been reviewed on average 5 times (1.88 standard deviation (SD)). Seven patients were discharged from the service due to non-attendance; 4 patients were discharged at 12-months follow-up and 3 at 24-months follow-up.

### Analysis of nurse practitioner-led CXL

Eighty-two CXL eyes (64.06%) were performed by a NP. Forty-two CXL eyes (32.81%) were performed by an ophthalmologist. For 4 eyes the surgeon was not identifiable, hence excluded from the analysis.

Best corrected visual acuity (BCVA) significantly improved at 24-months (*p* = 0.03387, Mann–Whitney U-Test) in patients treated by NP. K1 and K2 were reduced at 12-months follow-up in eyes treated by a NP (*p* = 0.04039, *p* = 0.01549, Mann–Whitney U-Test) but this reduction was not seen at 24-months. Anterior higher order aberrations were significantly elevated at 12-months follow-up (*p* = 0.04227, Mann–Whitney U-Test), but this elevation had reduced at 24-months follow-up and was no longer statistically significant. No significant changes in Kmax, pachymetry or posterior float were observed at 12- and 24-months (*p* > 0.05, Mann–Whitney U-Test).

**Fig. 2:**
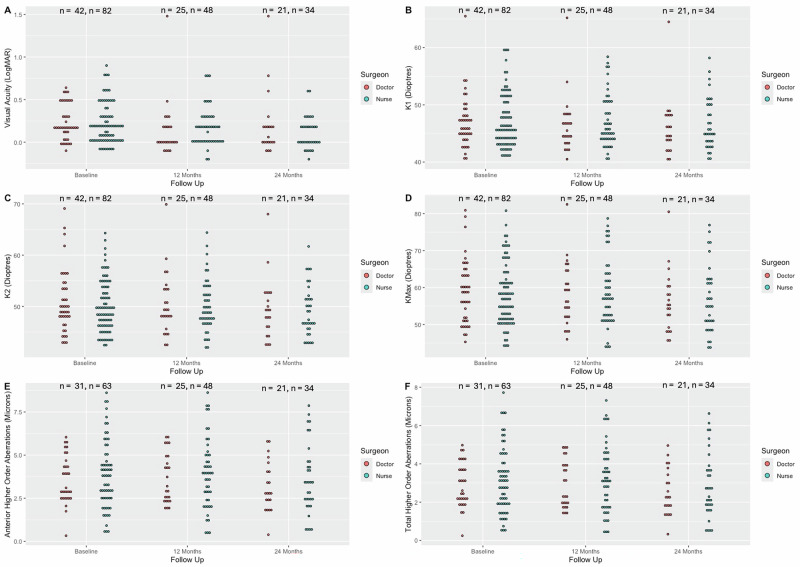
Dot plots comparing eyes that have had CXL performed by a nurse practitioner and an ophthalmologist (Doctor) at baseline, 12-months and 24-months. **A** Visual acuity (LogMAR), **B** K1 (dioptre), **C** K2 (dioptre), **D** Kmax (dioptres). **E** Anterior Higher-Order Aberrations (micrometres). **F** Total Anterior Higher Order Aberrations (micrometres).

Eyes treated by ophthalmologists showed no significant change in BCVA at 12- and 24-months follow-up (*p* > 0.05, Mann–Whitney U-Test, Fig. 2). No significant changes in K1, K2, Kmax or posterior float at 12- or 24-months follow-up (*p* > 0.05, Mann–Whitney U-Test). Pachymetry was significantly reduced at 12-months follow-up (*p* < 0.05, Mann–Whitney U-Test) but this change was no longer significant at 24-months follow-up (*p* > 0.05, Mann–Whitney U-Test, Fig. 2).

No significant differences in BCVA or corneal topography were found when comparing CXL procedures performed by NPs or ophthalmologists (*p* > 0.05, Mann–Whitney U Test, Table 2).

**Table 2:** Monitoring Values in the CXL group pre-CXL and at 12- and 24-months following CXL.

	Nurse-Led CXL	Doctor-Led CXL	*P* value
*Visual acuity (LogMAR)*			
*Baseline*	0.42 (0.00–0.85)	0.23 (0.17–0.30)	*P* = 0.3621
*12-Months*	0.17 (0.11–0.24)	0.13 (0.01–0.26)	*p* = 0.1102
*24-Months*	0.11 (0.04–0.17)	0.19 (0.03–0.35)	*p* = 0.7133
*K1 (Dioptre)*			
*Baseline*	47.18 (46.16–48.20)	47.00 (45.64–48.36)	*p* = 0.9012
*12-Months*	47.28 (45.99–48.57)	46.45 (44.53–48.36)	*p* = 0.38
*24-Months*	46.62 (45.10–48.14)	46.04 (43.88–48.19)	*p* = 0.5442
*K2 (Dioptre)*			
*Baseline*	50.05 (48.96–51.14)	50.88 (49.05–52.72)	*p* = 0.5811
*12-Months*	50.20 (48.79–51.61)	50.37 (48.01–52.73)	*p* = 0.9954
*24-Months*	49.21 (47.57–50.86)	49.44 (46.91–51.97)	*p* = 0.9585
*Kmax (Dioptre)*			
*Baseline*	64.40 (53.66–75.13)	58.79 (56.21–61.37)	*p* = 0.8183
*12-Months*	58.60 (56.00–61.19)	58.12 (54.82–61.42)	*p* = 0.9167
*24-Months*	56.78 (53.74–59.82)	56.06 (52.52–59.61)	*p* = 0.8285
*Pachymetry (Microns)*			
*Baseline*	450.87 (441.52–460.21)	463.24 (451.20–475.28)	*p* = 0.1218
*12-Months*	443.35 (431.33–455.38)	451.96 (439.64–464.28)	*p* = 0.1889
*24-Months*	454.94 (440.45 –469.43)	441.10 (398.74–483.47)	*p* = 0.9241
*Posterior Float (Microns)*			
*Baseline*	60.59 (54.10–67.08)	57.43 (48.53–66.33)	*p* = 0.6497
*12-Months*	60.67 (53.16–68.18)	61.16 (49.98–72.34)	*p* = 0.9536
*24-Months*	54.23 (43.13–65.34)	55.86 (41.53–70.19)	*p* = 0.6337
*Anterior Higher Order Aberrations (Dioptres)*			
*Baseline*	3.87 (3.39–4.36)	3.60 (3.11–4.09)	*p* = 0.6237
*12-Months*	3.95 (3.37–4.53)	3.68 (3.11–4.25)	*p *= 0.697
*24-Months*	3.60 (2.91–4.29)	3.19 (2.57–3.81)	*p* = 0.5229
*Total Higher Order Aberrations (Dioptres)*			
*Baseline*	3.14 (2.72–3.55)	2.90 (2.50–3.31)	*p* = 0.8312
*12-Months*	3.20 (2.71–3.69)	2.93 (2.44–3.42)	*p* = 0.7075
*24-Months*	2.90 (2.31–3.49)	2.53 (2.01–3.05)	*p *= 0.5115
*Sphere (Dioptres)*			
*Baseline*	−0.08 (−0.88 to 0.71)	0.04 (−0.89 to 0.97)	*p* = 0.5661
*12-Months*	0.18 (−1.08–1.43)	1.00 (−0.17 to 2.17)	*p* = 0.4665
*24-Months*	0.98 (0.08–1.87)	0.46 (−1.19 to 2.12)	*p* = 0.6621
*Cylinder (Dioptres)*			
*Baseline*	−3.42 (−3.95 to 2.90)	−4.09 (−5.02 to 3.16)	*p* = 0.2881
*12-Months*	−4.07 (−4.77 to 3.38)	−4.54 (−5.74 to 3.34)	*p* = 0.8358
*24-Months*	−3.92 (−5.01 to 2.83)	−4.12 (−5.40 to 2.84)	*p* = 0.75

Data presented as median (95% Confidence Intervals).

(Doctor = ophthalmologist).

Subjective cylinder increased following CXL (*p* = 0.0009378, *p* = 0.03314, Mann–Whitney U-Test). Subjective base sphere significantly increased at 12-months-follow-up but was no longer significant at 24-months. No differences in topographic cylinder or spherical equivalent were found (*p* > 0.05, Mann–Whitney U-Test). No significant differences in spherical or cylindrical refraction were found when comparing CXL procedures performed by NP or ophthalmologists (*p* > 0.05, Mann–Whitney U Test).

Three eyes that underwent CXL by a NP (3.66%) experienced stromal haze following CXL. Of these eyes, 1 eye had persistent corneal haze at 24-months follow-up. Six eyes (14.29%) that had CXL performed by an ophthalmologist developed stromal haze following CXL, with 2 eyes having persistent stromal haze at 24-months. Cases who developed corneal haze were immediately seen and transferred to the cornea consultants’ clinics where prednisolone acetate 0.5% eye drops was prescribed 4 times/day for an additional 4–6 weeks and the patients were followed-up in the respective consultant clinic thereafter. The difference in initial incidence of corneal haze was statistically significant (*p* < 0.05, Chi-squared test).

### Untreated group

All patients in this group had baseline refraction and corneal topography performed. Patients were reviewed on average 4 times (mean number of appointments = 3.71; 1.31 SD). Six eyes (4.05%) were found to have progression and were listed for corneal cross-linking. One patient declined treatment and was removed from analysis. Visual acuity within the remaining patients improved during the monitoring period (*p* = 0.03493, Table 3). No changes in corneal topography or subjective refraction were seen over the monitoring period (*p* > 0.05, Mann–Whitney U-Test).

**Table 3:** Values in the monitoring group at first appointment and at most recent review.

Value	Baseline	Most recent visit
*Visual Acuity (LogMAR)*	0.09 (0.03–0.15)	0.02 (−0.01 to 0.05)
*K1 (Dioptre)*	45.05 (44.43–45.67)	45.06 (44.42–45.70)
*K2 (Dioptre)*	47.98 (47.24–48.72)	47.90 (47.14–48.65)
*Kmax (Dioptre)*	52.50 (51.33–53.66)	55.59 (49.53–61.66)
*Pachymetry (microns)*	473.94 (465.42–482.47)	468 (457.69–478.31)
*Posterior Float (microns)*	44.58 (38.96–50.20)	40.26 (35.23–45.28)
*Higher Order Aberrations (Anterior)*	2.41 (2.14–2.69)	2.45 (2.17–2.72)
*Higher Order Aberrations (Total)*	1.95 (1.72–2.18)	1.99 (1.76–2.21)
*Sphere*	−0.52 (−1.00–−0.05)	0.06 (−0.51 to 0.63)
*Cylinder*	−2.67 (−3.14–−2.21)	−2.63 (−3.29 to −1.96)

Data presented as mean (95% Confidence Intervals).

## Discussion

This paper evaluates the outcomes of an AHP-led keratoconus service reviewing both untreated keratoconus patients and patients treated with CXL. Ophthalmology referrals comprise 10% of all outpatient referrals in the UK, the largest of any speciality [14]. This huge demand for ophthalmic services in the UK has escalated over the past decade with the COVID-19 pandemic increasing waiting list times for ophthalmic services [15, 16]. For corneal clinics, this means many keratoconus patients, both new referrals and follow-up patients, are waiting longer for monitoring clinic appointments increasing the risk of progression and sight loss. This also means that CXL patients have access to fewer follow-up appointments.

Tertiary eye units across the UK are utilising AHP’s more and more in specialist services with approximately half of the corneal services employing optometrists in extended roles [12]. Virtual optometry-led clinics increased corneal clinic capacity by approximately 500 appointments [17]. The virtual clinic in the study by Molero-Senosiain et al. only analysed the changes in corneal topography and best-corrected visual acuity and did not perform refraction or monitor patients following CXL. A single study analysing an in-person service found AHP’s monitoring keratoconus patients to have similar levels of clinical judgement with consultant-led keratoconus clinics [18]. Both studies monitor patients before CXL, but neither evaluates the ability of AHP’s to monitor patients following CXL. To our knowledge, this is the first paper to analyse the results of patients who were primarily reviewed by optometrists following CXL. Our study demonstrates safe monitoring of keratoconus patients pre- and post-crosslinking with timely decision making for proceeding to CXL and management of any complications.

Our results found that AHP-led service provided a standardised and thorough evaluation of patients at each clinic appointment. A thorough keratoconus assessment requires history taking, slit-lamp examination, topography and refraction to be completed. This typically requires a dual assessment by an ophthalmologist and optometrist because most refractive assessments in the UK are performed by optometrists. This dual assessment costs time transferring patients from one professional to another; therefore, this service model could streamline corneal services, ensuring clinic appointments are more time efficient. This efficiency could have a knock-on effect in increasing clinic capacity, allowing more patients to be seen.

Additionally, this is the first paper to our knowledge to look at the outcomes of patients who have undergone crosslinking by a NP. Interestingly, the best spectacle corrected visual acuity improved in the patients undergoing crosslinking performed by NP, with some topographical evidence of corneal flattening at 12 but not at 24 months. The ophthalmologist delivered CXL did not show statistically significant improvement in spectacle corrected visual acuity at 12 and 24 months. This may have been attributed to different surgeons performing CXL as well as more advanced and some younger patients in the cohort performed by ophthalmologists. Previous studies have found evidence of corneal flattening and reduction in higher-order aberrations [19–21].

Many keratoconus patients have functional vision with contact lenses. It is well known that contact lenses, especially RGP lenses, can warp the cornea and affect keratometry. Patients are required to remove RGP lenes for two weeks prior to assessment. This is important for refractive surgery and biometry for cataract surgery but may not be so for CXL if the same protocol of removing contact lenses immediately prior to topography is maintained at each visit. Patients who were attending for monitoring were reluctant to stay off contact lenses, even in one eye, for every follow-up visit as this affected their ability to work and drive.

Complications arising from corneal cross-linking vary in their incidence. Corneal haze is the most commonly reported complication of CXL with reported incidence at 1 year varying between 2–19% [22–24]. Our incidence of corneal haze in AHP-led CXL cases matches previously reported incidences. These complications were commonly reported within the first 3 months of follow-up; however, subsequent documentation does not consistently mention whether this initial corneal haze resolved. Initial corneal haze is commonly differentiated from permanent long-term corneal haze due to differences in the underlying aetiology. The timely interference with increasing steroids to manage corneal haze can influence the outcome [25]. In our study, prompt diagnosis of corneal haze by the optometrist lead to successful treatment in majority of cases. Other reported complications in the literature include microbial keratitis, endothelial damage, herpes simplex reactivation, treatment failure and continued flattening [26]. None of these complications were found in our treated patients.

A limitation of this study is the lack of uniform follow-up appointments for patients resulting in missing datapoints at different time intervals. This study analyses real clinical data and patients were reviewed at different intervals based upon their individual risk of progression. Factors which could affect progression include age at diagnosis, baseline topography and refraction. Patients at high risk of progression were be reviewed more frequently. In contrast, stable patients at low risk of progression may be reviewed only annually. This could introduce selection bias into the data as high-risk patients are more likely to have clinical data available at regular intervals in comparison to low-risk patients. However, from a clinical perspective, this new clinic model needs to care for a spectrum of keratoconus patients, including recognising patients at high risk of progression and monitoring them more closely. Further follow-up sub-group analysis of high-risk vs low-risk patients could validate whether both patient groups are being monitored appropriately. This limitation can be addressed by establishing follow-up protocols for low-risk and high-risk patients but in the context of the NHS service provision, the disparity between the follow-up date indicated by the clinician and the actual date the patient is sent for, introduces variations that are difficult to control in the real world setting.

There is also a potential selection bias where more advanced keratoconus patients may have been streamed directly to consultant-led clinics or their crosslinking have been performed by the doctor. These advanced patients, especially those requiring corneal grafts, are missing from the dataset. This may have been a contributing factor to more cases of haze developing in the crosslinked group done by the doctor. Larger prospective studies need to be performed to validate these points. Nevertheless, the aim of the AHP-led clinic was to assess more “routine” keratoconus patients therefore keratoconus patients requiring corneal grafts were not in the population of interest. Similarly, the selection bias for patients requiring general anaesthesia, hence for the procedure to be performed by an ophthalmologist in the operating theatre, would exclude these individuals from the APH-led CXL service. This will always remain a factor with the APH-led service but the numbers are few therefore will not detract from the importance of the APH-led service.

The other factors affecting attendance were clinic cancellations and failure to keep appointments. Many of our patients referred in 2019 and 2020 had their appointments cancelled or were lost to follow-up due to the COVID-19 pandemic [15]. Failure to attend clinics may also be attributed to the age of the patients who are young and of working age. Younger patients have higher rates of hospital appointment non-attendance, with work and childcare commitments highlighted as potential causes [27]. Paediatric keratoconus patients depend on parents or carers to bring them to clinic and their attendance is affected by factors relating to their responsible caretaker. Increasing digital appointment reminders may reduce non-attendance rates and help any subsequent data collection [28]. Examining non-attendance at other clinic models such as virtual clinics could also potentially support a virtual and in-person model, where keratoconus patients who struggle to routinely attend lengthy face-to-face appointments attend virtually with those showing evidence of topographic progression invited to face-to-face assessment. Though virtual clinics improve efficiency, whether they will improve the attendance of younger working patients and of those who depend on carers to bring them to appointments, will need to be tested.

Despite these limitations our study shows that the AHP-keratoconus service was successful in monitoring keratoconus patients with timely identification of patients requiring CXL, preforming the procedure and conducting follow up examinations enabling prompt identification of any complications. For this model to be successful, robust training, accreditation and supervision of the AHPs; with regular audits and clear escalation pathway with prompt access to ophthalmologists for advice and guidance, must be put in place.

## Summary

### What was known before

- Keratoconus requires frequent monitoring using multiple different techniques to assess for progression and need for therapeutic intervention. This creates a large burden on corneal clinics and is difficult to balance with the need to see patients in corneal clinic with sigh-threatening corneal pathology.
- In the United Kingdom, there is increasing utilisation of Allied Health Professionals in extended clinical roles, expanding their skillset to assess and treat more complex pathologies.
- Virtual clinics ran by optometrists to assess keratoconus patients have been shown to be efficacious.

### What this study adds

- Allied health professional-led keratoconus clinics safely monitor patients following collagen cross-linking. Complications were detected early, and vision improved in patients treated by nurse practitioners.
- Collagen Cross-linking performed by nurse practitioners is safe with no increase in observed complications.
- Patients monitored face-to-face by optometrists effectively highlighted patients who required collagen cross-linking and safely monitored the remaining stable keratoconus patients.

## Data Availability

Data available upon request.
